# Bioaccumulation and Trophic Transfer of Mercury and Selenium in African Sub-Tropical Fluvial Reservoirs Food Webs (Burkina Faso)

**DOI:** 10.1371/journal.pone.0123048

**Published:** 2015-04-13

**Authors:** Ousséni Ouédraogo, John Chételat, Marc Amyot

**Affiliations:** 1 Groupe de recherche interuniversitaire en limnologie, Département de sciences biologiques, Université de Montréal, Montréal, Québec, Canada; 2 Environment Canada, National Wildlife Research Centre, Carleton University, Ottawa, Ontario, Canada; University of Girona, SPAIN

## Abstract

The bioaccumulation and biomagnification of mercury (Hg) and selenium (Se) were investigated in sub-tropical freshwater food webs from Burkina Faso, West Africa, a region where very few ecosystem studies on contaminants have been performed. During the 2010 rainy season, samples of water, sediment, fish, zooplankton, and mollusks were collected from three water reservoirs and analysed for total Hg (THg), methylmercury (MeHg), and total Se (TSe). Ratios of δ^13^C and δ^15^N were measured to determine food web structures and patterns of contaminant accumulation and transfer to fish. Food chain lengths (FCLs) were calculated using mean δ^15^N of all primary consumer taxa collected as the site-specific baseline. We report relatively low concentrations of THg and TSe in most fish. We also found in all studied reservoirs short food chain lengths, ranging from 3.3 to 3.7, with most fish relying on a mixture of pelagic and littoral sources for their diet. Mercury was biomagnified in fish food webs with an enrichment factor ranging from 2.9 to 6.5 for THg and from 2.9 to 6.6 for MeHg. However, there was no evidence of selenium biomagnification in these food webs. An inverse relationship was observed between adjusted δ^15^N and log-transformed Se:Hg ratios, indicating that Se has a lesser protective effect in top predators, which are also the most contaminated animals with respect to MeHg. Trophic position, carbon source, and fish total length were the factors best explaining Hg concentration in fish. In a broader comparison of our study sites with literature data for other African lakes, the THg biomagnification rate was positively correlated with FCL. We conclude that these reservoir systems from tropical Western Africa have low Hg biomagnification associated with short food chains. This finding may partly explain low concentrations of Hg commonly reported in fish from this area.

## Introduction

Aquatic environments are sinks for most contaminants including trace metals and metalloids. At low concentrations in the aquatic environment, some of these contaminants have the potential to biomagnify through the food chain leading to levels of concern in top predators. The West-African landscape of today is characterized by the presence of many small water reservoirs used for multiple purposes including livestock watering, irrigation, flood protection, groundwater recharge, and human drinking water [1]. Previous studies on elemental biomagnification in Africa have mainly focused on mercury (Hg) and were conducted in large lakes such as those of the Great Lakes region from Eastern Africa [2,3,4]. Little is known about Hg biomagnification in smaller systems such as fluvial reservoirs from Western Africa. Furthermore, the biomagnification potential of metalloids such as selenium (Se) has received little attention in Africa. Se is an essential micronutrient showing a narrow margin between nutritionally optimal and potentially toxic concentrations. Given conflicting findings in the literature on whether Se biomagnifies through aquatic food webs (e.g., [5,6,7]), research on Se in African ecosystems is needed. Further, due to the increasing evidence of antagonistic interactions between Hg and Se [8,9,10,11,12], investigation on Hg biomagnification and exposure risk assessment in an ecosystem should consider Se availability. Se to Hg molar ratios have been recently used in risk assessment as a proxy to evaluate the degree of Se protection against Hg toxicity and bioaccumulation in biota. Higher selenium concentration than Hg (Se to Hg molar ratio > 1) might reduce Hg concentration and toxicity in biota [13,14,15,16].

In a previous survey in Burkina Faso [17], we reported relatively low levels of Hg and Se in fresh water and fish. In the present study, we focus on characterizing the aquatic food web structure and the bioaccumulation and biomagnification of a trace metal (Hg) and a metalloid (Se) deposited into three fluvial reservoirs in Burkina Faso, using stable isotope ratios of nitrogen (δ^15^N) and carbon (δ^13^C). Carbon and nitrogen stable isotope ratios have been successfully used to analyze trophic relationships and food web structures in lake ecosystems [18,19]. In aquatic environments, pelagic and benthic algae often show distinctive carbon stable isotope signatures as a result of different fractionation during carbon fixation [20,21]. Benthic algae generally exhibit less ^13^C fractionation during carbon fixation than phytoplankton resulting in enriched δ^13^C ratios in benthic zones. Further, δ^13^C values are relatively unaffected by trophic transfer (< 1‰ fractionation between a predator and its prey) [20,22]. Thereby, it is commonly used to provide information about the sources of energy to food webs [23]. In tropical systems, there are several potential carbon sources such as macrophytes, terrestrial detritus, benthic algae, and phytoplankton, and high algal growth rates may reduce carbon isotope discrimination [4]. In addition, a stepwise trophic level enrichment in δ^15^N of 3–4‰ (mean = 3.4 ‰) has been reported [19,24], allowing the use of δ^15^N ratios to trace contaminant biomagnification [18,25,26]. Food chain length can be estimated with δ^15^N ratios when values for bottom trophic levels and top predators are measured [27,28]. Food chain length (FCL) is a measure of the number of trophic links between primary producers and the top predator in an ecosystem. It has long been recognized as a fundamental ecosystem attribute [29] and likely plays a role in the contaminant bioaccumulation in top predators [18].

The first objective of this study was to describe the structure of freshwater food webs in Burkina Faso reservoirs using carbon and nitrogen stable isotopes. The second objective was to characterize Hg and Se bioaccumulation and biomagnification in these food webs and to examine the role of biological and environmental factors responsible for Hg and Se concentrations in fish. We then compared FCL and THg biomagnification in fluvial reservoirs to other aquatic ecosystems that have been studied in Africa (mainly large lakes) to determine if there is system-specific variation in these characteristics. We hypothesized that short food chains in the reservoirs may explain the low THg biomagnification in those systems.

## Materials and Methods

### 2.1. Study sites

**Fig 1:**
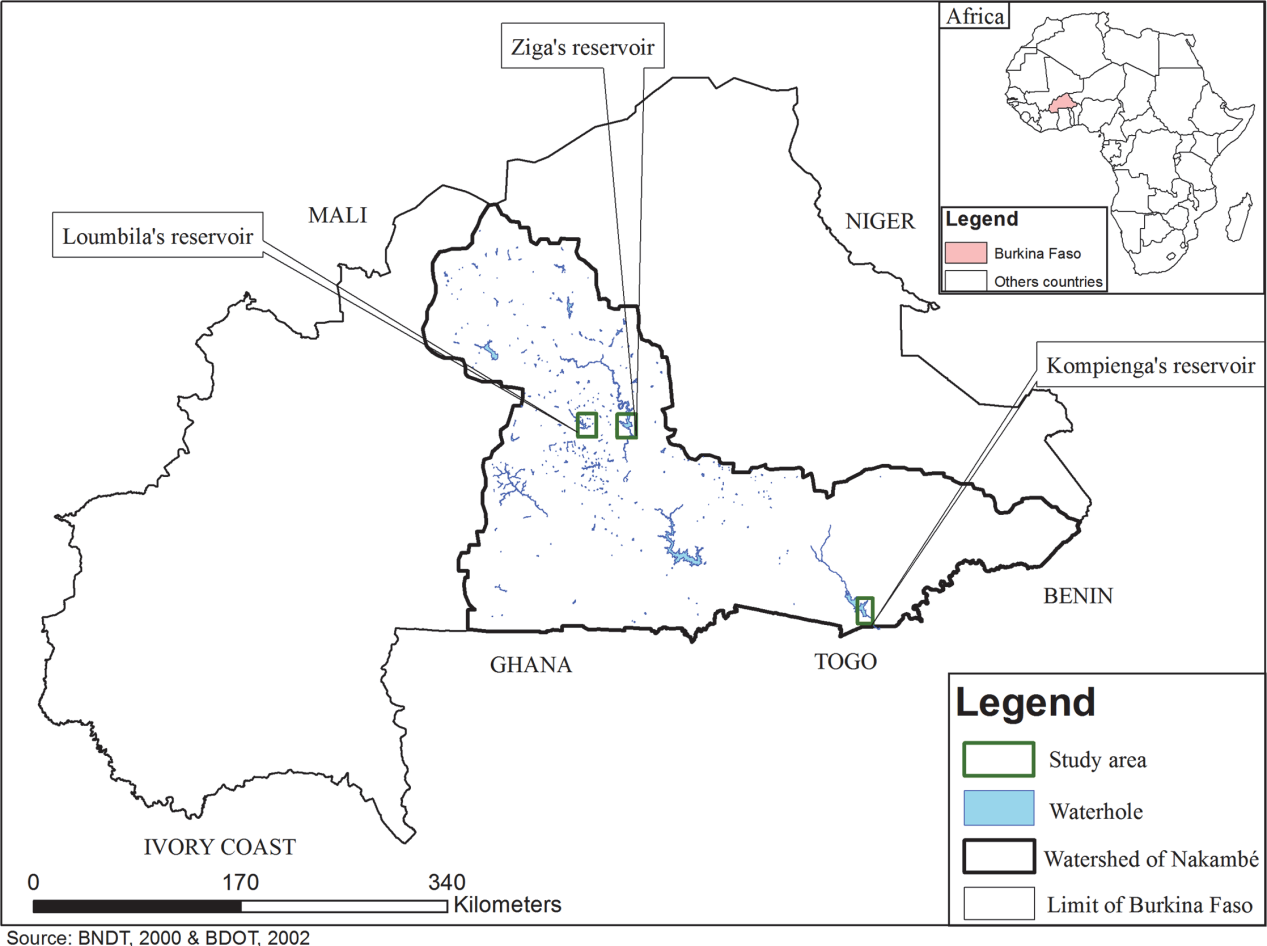
Map of study areas showing locations of the three reservoirs in Burkina Faso. Source: Base Nationale de Données Topographiques (BNDT), 2000 and Base de Données d’Occupation des Terres (BDOT), 2002 of Burkina Faso.

The three reservoirs were located in the central part of Burkina Faso in the Nakambe River catchment area (Fig 1). The first one, Loumbila dam (12° 29’N, 1° 24’ W), is a small man-made reservoir built in 1947, used to provide water for people, livestock and agriculture. It has an average surface area of 1500 ha and a mean depth of 6.5 m. The second system, Ziga dam (12° 30’ N, 1° 4’ W), is an 8,000 ha reservoir that was built twenty years ago for drinking water supply. The third reservoir, Kompienga (11° 5’ N, 0° 41’ W), was built in 1988 and is one of the two largest hydroelectric reservoirs in the country with a surface area ranging from 16,000 to 20,000 ha. All sites had warm waters (26–30°C), low levels of dissolved organic carbon (DOC) (average of 2 mg/L), were not thermally stratified during the sampling period (S1 Fig) and had a circumneutral pH (S1 Table). Trace element levels in water were found to be similar in the three reservoirs to those reported in a previous field survey [17]. Differences in watershed use and reservoir size (depth, area) as well as the age of the reservoir led to the choice of these sites to investigate bioaccumulation and biomagnification patterns, since they cover the range of reservoirs found in Burkina Faso. No specific permissions were required for sampling Loumbila and Ziga reservoirs. For Kompienga, a permit was issued by the Société Nationale d’Électricité du Burkina.

### 2.2. Field sampling

During the 2010 rainy season (July-August, rising water) in Burkina Faso, water, sediment, zooplankton, molluscs and fish were collected from the three Nakambe catchment reservoirs.

#### 2.2.1. Water collection

Ultra-clean protocols for trace metals [30] were employed to collect water. In each reservoir, water was collected on one occasion at the near shore station (littoral zone) and open water station (pelagic zone) at a depth of 0.5 m from the sediment surface where THg and MeHg concentrations were potentially higher [31]. Detailed protocol for water collection is given in S1 Protocol.

#### 2.2.2. Zooplankton sampling

Zooplankton were sampled for trace metal and stable isotope analyses with a 0.25 m diameter net of 100 μm mesh size in the pelagic zone of each lake by vertical hauls. A large volume of water was filtered to collect enough biomass for analyses. Samples were transferred into clean Teflon containers, placed in double Ziploc bags at -20°C, freeze-dried and stored in double Ziploc bags at 4°C until analysis.

#### 2.2.3. Benthos and fish sampling

Fish samples were bought from local fishermen in a unique fishery store located near each reservoir dam. Twenty to thirty fish were obtained for each fish species. After taking body measurements, sections of dorsal muscle tissue were taken for analyses of trace elements and stable isotope ratios. Dorsal muscle samples were kept in polyethylene bags, frozen at -20°C, freeze-dried and shipped to Canada for laboratory analysis. Approximately 350 individual fish were collected and included the five fish species most consumed by the local population, namely *Oreochromis niloticus* (Nile tilapia, Cichlidae, detritivore), *Clarias anguillaris* (Catfish, Clariidae, omnivore), *Bagrus bajad* (Bagridae, piscivore), *Auchenoglanis occidentalis* (Claroteidae, invertebrates-feeders), and *Schilbe intermedius* (Schilbeidae, invertebrates-feeders, piscivore). Additional species such as *Lates niloticus* (Nile perch, Centropomidae, piscivore), *Synodontis membranaceus* (Mochokidae, planktivore), and *Hydrocynus forskalii* (Alestidae, piscivore) were also collected. Dietary preferences of fish were identified based on stomach content analysis [32,33]. All fish were bought from local fishermen who captured the fish by traditional methods. Therefore, we did not need an approval by an Institutional Animal Care and Use Committee. Profundal bivalves (Iridinidae) were sampled using an Ekman grab and gastropods were hand removed by fishermen from their nets. All invertebrates were prepared in the same manner as fish for trace metal and isotopic analyses. This field study did not involve endangered or protected species.

### 2.3. Laboratory analysis

#### 2.3.1. Water analyses

Vertical profiles of water temperature (°C), dissolved oxygen concentration (%), pH, and conductivity (μS/cm) were measured at 0.5 m intervals from each site using a YSI-650 DMS multiprobe. Oxygen and pH calibrations were completed every sampling day. Major anions were analysed by ion chromatography and cations by atomic absorption spectrometry.

THg and MeHg analysis in water samples (filtered and unfiltered) was performed by cold vapor atomic fluorescence spectrometry (CVAFS). TSe concentrations in water samples were determined by hydride generation atomic fluorescence spectrometry (HG-AFS). For more details, see S1 Protocol.

#### 2.3.2. Total mercury analysis

Solid tissues from aquatic organisms (fish, zooplankton, bivalves, gastropods) were analyzed for THg using a direct mercury analyzer (DMA 80, Milestone Inc., Pittsburgh, PA), in which samples were combusted at 750°C and mercury vapors were retained on a gold trap for analysis by cold vapor atomic absorption spectrometry (CVAAS). DMA threshold analysis was between 0.12 and 600 ng and detection limit was 0.05 ng THg/sample. The certified reference materials TORT-2 (lobster hepatopancreas, National Research Council, Canada) and DORM-3 (National Research Council, Canada) were used for quality control (results are provided in S2 Table).

#### 2.3.3. Methylmercury analysis

For MeHg analysis in solid tissues (fish, zooplankton, gastropods and bivalves), 10 to 50 mg of dried homogenized tissue was digested in 5 mL of 4M HNO_3_ at 55°C for 16 h. Digested samples then underwent aqueous-phase ethylation followed by gas chromatography separation with CVAFS detection (Tekran 2500). Analytical accuracy was checked by analysis of TORT-2 after each 10 samples (S2 Table). The method detection limit (MDL) based on three times the standard deviation of 10 blanks was 0.02 ng/L and the average coefficient of variation (standard deviation/mean) for field triplicate determinations was 13%.

#### 2.3.4. Selenium analysis

For TSe determination in solid samples (fish, zooplankton, gastropods and bivalves), 20 to 50 mg of solid tissue was digested in a microwave with a mixture of HNO_3_ and H_2_O_2_ based on a method developed by Corns et al.[34] to extract elements from a solid matrix. An aliquot of 4 mL was then taken and treated using the same protocol used for aqueous samples.

The analytical quality for TSe was controlled using certified reference materials DORM-3 and TORT-2 from the National Research Council of Canada (S2 Table). Efficacy of Se (VI) conversion to Se (IV) was checked by using a solution of Se (VI) that was analyzed together with the samples. Procedural blanks contained 21 ± 8 ng TSe /L(n = 8). The MDL was 22 ng/L (aqueous Se) and 0.022 μg/g dry weight (d.w.) for solid samples. Conversion of 200 ng/LSe (VI) to Se (IV) averaged 109% ± 9.

#### 2.3.5. Stable isotope analyses

Stable isotope analyses were conducted at the GEOTOP research centre of the Université du Québec à Montréal (UQÀM). Prior to analysis, sediment, freeze-dried fish and invertebrate tissue samples were homogenized into a powder. Zooplankton samples were analyzed in bulk due to their small size. Small sub-samples of ground tissues were weighed in tin cups and analyzed on a Micromass Isoprime isotope ratio mass spectrometer in continuous flow mode coupled to an Elementar Vario Micro Cube elemental analyzer. Analytical precisions for δ^13^C and δ^15^N measurements were 0.1‰ and 0.2‰ respectively.

### 2.4. Data analysis

#### 2.4.1. Food web structure analysis

The δ^13^C value is used to investigate whether organisms obtain their carbon from the pelagic zone or the littoral benthic zone. More positive δ^13^C values likely represent consumption of littoral benthic carbon while more negative values likely represent pelagic carbon use. High variability has been documented within and among systems in the δ^15^N and δ^13^Cvalues at the base of the food web from which organisms draw their nitrogen and carbon [35,36]. For example, the range in δ^13^C observed within and across lakes can be driven by high spatial and temporal variability in phytoplankton biomass and growth rate, with very high biomass or growth rates tending to lead to reduced isotopic discrimination and higher δ^13^C values. As a result, it is standard practice for among system comparisons to use δ^15^N signatures of primary consumers (rather than primary producers) as baseline indicators for estimating trophic position because their large body size and greater longevity result in less seasonal changes in δ^15^N [35,37,38]. Previous research [35] has demonstrated a covariance of δ^15^N between primary consumers (e.g. Unionid mussel) and higher level consumers such as walleye and yellow perch from the same lake. So, despite the fact that Unionids were not a dietary item of walleye and yellow perch, δ^15^N of this primary consumer was useful for correcting the signature of higher level consumers to provide a more accurate reflection of their trophic position. Based on this recommendation and because of multiple potential organic matter sources available in the tropical food webs (macrophytes, terrestrial detritus, benthic algae, phytoplankton) [37], the average δ^15^N values of all sampled primary consumers (including mussel, gastropods, zooplankton) were used as a baseline to standardize the δ^15^N values of fish collected in these reservoirs. Adjusted δ^15^N (δ^15^N_adj_) values were calculated using the approach of Vander Zanden and Fetzer [28].

$$\delta^{15}N_{\mathrm{adj}.}=\delta^{15}N_{\mathrm{organism}}-\delta^{15}N_{\mathrm{primary consumers}}$$

Trophic position (TP) of consumers was then calculated using δ^15^N_adj_, as follows:

$$\mathrm{TP}=\lambda +\delta^{15}N_{\mathrm{adj}.}/\Delta_{n}$$

where Δ_n_ represents the isotopic enrichment per trophic level assumed to be 3.4‰ [38,39], λ is the trophic position of the baseline indicator. The primary consumers were assumed to have a trophic position of 2.

For each system, FCL was calculated according to Vander Zanden and Fetzer [28] as:

$$\text{FCL}=\left(\delta^{15}N_{\mathrm{top predator}}-\delta^{15}N_{\mathrm{baseline}}\right)/\Delta_{n}-\lambda$$

The site-specific baseline δ^15^N value was estimated using the mean δ^15^N of all primary consumer invertebrates from each reservoir [28].

#### 2.4.2. Biomagnification of Hg and Se analysis

The biomagnification rates of Hg and Se were investigated in the food web of the three reservoirs using the following equation of [40]:

$${\text{log}}_{10}C_{M}=A\times \delta^{15}N+B$$

where *C*_M_ (the concentration of a given metal) represents THg, MeHg or TSe concentrations, A is the slope of the equation, also referred to as the rate of biomagnification or trophic magnification slope (TMS), and B is the intercept. TMS estimates the average increase in metal(loid) concentration per unit δ^15^N.

A trophic magnification factor (TMF) is the average factor change in metal(loïd) concentration between two trophic levels [41,42,43]. TMFs were calculated as the antilogarithm of *m* (TMF = 10^*m*^), where *m* is the slope of the regression of log_10_-transformed-metal(loïd) vs. δ^15^N (or TMS), multiplied by 3.4 (the average increase in δ^15^N per trophic level, [39]). A TMF above 1 indicates an increase in metal(loïd) concentration with increasing trophic position (i.e. food web biomagnification) whereas, a TMF < 1 indicates trophic dilution [41,44]. In addition to the determination of Hg and Se biomagnification rates, potential protective effects of Se on Hg bioaccumulation and toxicity were assessed by the regression between log (C_TSe_/C_Hg_) and trophic position (δ^15^N_adj_) [45].

#### 2.4.3. Comparison of THg biomagnification and food chain length in fluvial reservoirs with other African water bodies

The food web biomagnification of THg (measured as TMF; see section 2.4.2) and the FCL of the three fluvial reservoirs were compared with 14 other measurements for aquatic ecosystems in Africa. We made this comparison to place the biomagnification results for the reservoirs in a broader African context and to test the hypothesis that these systems have low Hg biomagnification because of short FCLs. The other aquatic ecosystems were great lakes (n = 8), other lakes (n = 5) and a freshwater river estuary (n = 1). For most systems, the biomagnification metrics were calculated from a log THg vs. δ^15^N regression for fish only, with the exception of three sites (Malawi, Bosomtwe and Abrewe) where invertebrates were also included.

For each system, FCL was calculated according to Vander Zanden and Fetzer [28] as indicated in the methods section 2.4.1. The fish species with the highest mean δ^15^N in each study was used as the top predator. The site-specific baseline δ^15^N value was estimated using the mean of all data available for primary consumer invertebrates from each study [28], with the exception of two sites where benthic algae and phytoplankton were used. A list of the specific biota used in the FCL calculations is provided in S3 Table. The number of fish species examined in each study was tabulated as a second descriptor of the food web.

#### 2.4.4. Statistical analysis

All the results are expressed as a mean ± standard error. Statistical analyses were performed with R software (version R-2.11.1) (http://www.r-project.org/). Prior to linear regression analyses, normality and homoscedasticity were checked using the Shapiro-Wilk test and Bartlett test of homogeneity of variances, respectively.

One way analysis of variance (ANOVA_s_) with the Bonferroni comparison was used when comparing multiple groups and *T*-test when comparing two independent groups in metal(loïd) concentrations, mean δ^13^C or δ^15^N values and mean trophic position of fish species among sites. If ANOVA assumptions could not be met, nonparametric tests were used: a Kruskal-Wallis test was applied for comparing multiple independent groups and Wilcoxon test when comparing two independent groups. The significance level for all tests was p ≤ 0.05.

Within each reservoir, simple linear regressions between log_10_-tranformed metal(loïd) or *C*_TSe/_ *C*_*T*Hg_ concentrations in fish and their trophic position (using δ^15^N) were run to evaluate the potential for contaminant biomagnification (TMS and TMF) in food webs.

A stepwise multiple regression analysis (using forward selection) was done within each reservoir to identify which variable or combination of fish size (total length), fish trophic position (δ^15^N) and fish carbon signature (δ^13^C) best predict the bioaccumulation of Hg or of TSe within and among species.

A correlation analysis (using Spearman rho coefficients because of non-linearity and non-normality in the data) was conducted to examine the influence of water body size (surface area, maximum depth), number of fish species, and FCL on the biomagnification of THg in African water bodies. In addition, the THg concentration of a commonly sampled fish species (the detrivore *O*. *niloticus*) was included for comparison of bioaccumulation among systems.

## Results

### 3.1 Food web structure of the three freshwater reservoirs

**Fig 2:**
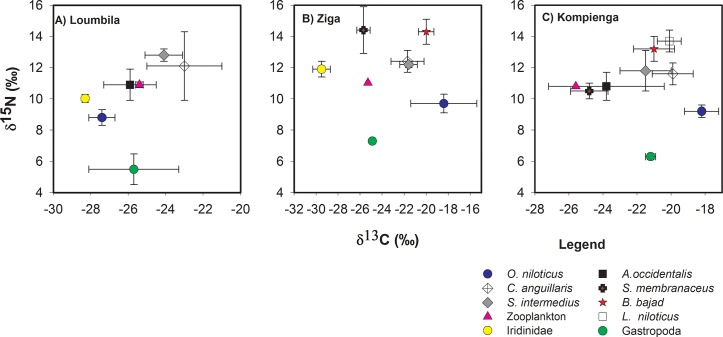
Food web structure of three freshwater reservoirs from Burkina Faso. The ratio of δ^15^N, indicating trophic position, and δ^13^C indicating dietary carbon source of biota in the freshwater reservoirs. Error bars = ± 1 standard deviation.

Scatterplots of δ^15^N against δ^13^C of all biota show both the carbon sources and trophic position for all fish species (Fig 2). The fish were supported by a range of carbon sources as indicated by δ^13^C values of -22 to -28‰ in Loumbila (Fig 2A), -16 to -30‰ in Ziga (Fig 2B) and -18 to -26‰ in Kompienga (Fig 2C). Nevertheless, the carbon isotope fractionation (δ^13^C) varied significantly across reservoirs for *O*. *niloticus* and for *C*. *anguillaris* (Kruskal-Wallis test; p < 0.05). *O*. *niloticus* had more negative δ^13^C values (-27.4 ‰) from Loumbila than those from the two others reservoirs (-18 ‰). Similarly,*C*. *anguillaris* from Kompienga showed less negative δ^13^C values compared to the other reservoirs. In addition, *C*. *anguillaris* from Loumbila particularly showed a range of δ^13^C values (-25 to -21‰) which suggests variable food sources for this species.

The mean trophic position of fish in the reservoirs ranged from 1.9 to 3.7 (S4 Table). The detritivore *O*. *niloticus* had the lowest δ^15^N values of all fish species in Loumbila (8.8 ± 0.5‰), Ziga (9.7 ± 0.5‰) and Kompienga (9.2 ± 0.4‰) reservoirs. Fish species at the top of the food web were *S*. *intermedius (*δ^15^N = 12.8 ± 0.4‰),*S*. *membranaceus (*δ^15^N = 14.4 ± 1.5‰) and *L*. *niloticus* (δ^15^N = 13.7 ± 0.7‰) for Loumbila, Ziga and Kompienga, respectively.

Omnivory was considered to be significant when the range of δ^15^N values for a species at a specific site exceeded Δ_n_ (3.4 ‰), the isotopic enrichment per trophic level [46,47]. Using this definition, 25% of fish species were omnivores on average at each site. When using alternate values of Δ_n_ ranging from 2.7 to 4.1 (i.e. 3.4 ± 2.0%), no change was found in the number of omnivores. FCL was slightly higher in the Kompienga reservoir (3.7 ± 0.2) than in Loumbila (3.4 ± 0.1) and Ziga (3.3 ± 0.4) (Table 1).

**Table 1:** A comparison of THg biomagnification rates in different types of African water bodies.

**Water body**	**Type**	**Country**	**Surface Area (km** ^2^ **)**	**Maximum depth (m)**	**TMS**	**TMF**	**# of fish species**	**Food chain length**	***O*. *niloticus* THg(μg/g ww)**	**Reference**
Victoria (Napoleon Gulf)	great lake	Uganda	68,000	69	0.16	3.5	6	3.4	0.006	Campbell et al. 2003
Victoria (Napoleon Gulf)	great lake	Uganda	68,000	69	0.20	4.8	13	3.3	0.013	Poste et al. 2012
Victoria (Winham Gulf)	great lake	Kenya	68,000	69	0.17	3.8	8	3.4	0.010	Campbell et al. 2003
Victoria (Thruston Bay)	great lake	Uganda	68,000	69	0.28	9.0	7	3.7	0.012	Campbell et al. 2004
Victoria (Murchison Bay)	great lake	Uganda	68,000	69	0.13	2.8	9	3.1	0.014	Poste et al. 2012
Albert	great lake	Uganda	5,300	58	0.26	7.7	21	3.8	—-	Campbell et al. 2005, Lavoie et al. 2013
Malawi	great lake	Malawi	29,600	706	0.20	4.8	40	3.9	—-	Kidd et al. 2003
Tanganyika	great lake	Tanzania	32,900	1,470	0.22	5.6	36	4.6	—-	Campbell et al. 2008
Saka	lake	Uganda	0.15	12	0.14	3.0	11	3.3	0.003	Campbell et al. 2006
Nkuruba	lake	Uganda	0.03	38	0.14	3.0	3	3.2		Campbell et al. 2006
Chad	lake	Chad	1,350	11	0.21	5.2	14	3.1	0.007	Kidd et al. 2004
Ziway	lake	Ethiopia	490	9	0.13	2.8	4	2.7	0.011	Tadiso et al. 2011
Bosomtwe	lake	Ghana	49	78	0.13	2.8	4	3.0	—-	Poste et al. 2008
Abrewe	river estuary	Ghana	—-	—-	0.21	5.2	1	3.2	—-	Poste et al. 2008
Loumbila	fluvial reservoir	Burkina Faso	2	6.6	0.13	2.9	4	3.4	0.006	this study
Ziga	fluvial reservoir	Burkina Faso	80	9	0.15	3.3	5	3.3	0.012	this study
Kompienga	fluvial reservoir	Burkina Faso	160–200	25	0.23	6.5	6	3.7	0.011	this study

The trophic magnification slopes (TMS) and trophic magnification factors (TMF) are based on linear regressions of log THg concentration vs. δ^15^N for fish only, with the exception of three sites (Malawi, Bosomtwe and Abrewe) where invertebrates were included. The number of fish species examined in each study and food chain length (see methods for calculation details) are provided as metrics of food web structure. The average THg concentration of a commonly sampled fish species (the detrivore *O*. *niloticus*) was included to compare among-site variation in mercury bioaccumulation. Full references for some of the studies are found in the supplemental information.

### 3.2. Factors influencing Hg and Se concentrations in fish

THg, MeHg and TSe concentrations in fish reported in the present study (Table 2) were low considering the World Health Organization guideline of 0.5 μg THg/g wet weight (w.w.) to protect groups vulnerable to mercury toxicity [48] and the TSe threshold of 3 μg/g (w.w) [49]. Levels of MeHg in water (range: 0.04–0.20 ng/L) and of THg in sediments (range: 8–27 ng/g dw) were low in all reservoirs (S1 Table). These concentrations were in the same range as reported and discussed in our previous survey [17].

**Table 2:** Mean (±sd) total length, THg, MeHg, total selenium and molar ratio TSe/THg, %MeHg, δ^15^N (‰), δ^13^C (‰) and trophic position (TP) of fish from three freshwater reservoirs (Burkina Faso).

**Reservoir/Organism**	**n1**	**TL (mm)**	**THg (μg/g w.w.)**	**TSe (μg/g w.w.)**	**Molar TSe/THg**	**Molar TSe/MeHg**	**n2**	**MeHg (μg/g w.w.)**	**% MeHg**	**δ** ^15^ **N (‰)**	**δ** ^13^ **C (‰)**	**TP**
**Loumbila**												
*O*. *niloticus*	32	147 ± 14	0.006 ± 0.003	0.14 ± 0.02	78 ± 35	23.0 ± 9.0	5	0.006 ± 0.003	91 ± 30	8.8 ± 0.5	- 27.4 ± 0.7	2.2 ±0.1
*A*. *occidentalis*	28	193 ± 17	0.023 ± 0.010	0.09 ± 0.06	8 ± 3	3.6 ± 1.8	5	0.017 ± 0.004	69 ± 20	10.9 ± 1.0	- 25.9 ± 1.4	2.9 ± 0.3
*C*. *anguillaris*	28	264 ± 80	0.064 ± 0.060	0.18 ± 0.06	8 ± 7	4.0 ± 1.7	9	0.040± 0.030	91 ± 18	12.1 ± 2.2	- 23.0 ± 2.0	3.2 ± 0.6
*S*. *intermedius*	14	145 ± 10	0.230 ± 0.070	0.20 ± 0.03	2 ± 0	1.3 ± 0.1	3	0.185 ± 0.064	63 ± 8	12.8 ± 0.4	- 24.1 ± 1.0	3.4 ± 0.1
Zooplankton	bulk		0.020		not analysed	not analysed			not analysed	10.9 ± 0.18	-25.4 ± 0.2	2
Iridinidae	4		0.024 ± 0.005	0.40 ± 0.01	not analysed	not analysed	4	0.025±0.001	not analysed	10.0 ± 0.2	- 28.3 ± 0.1	2
Gastropoda	5		0.026 ± 0.015	0.20 ± 0.04	not analysed	not analysed	5	0.013 ± 0.004	not analysed	5.5 ± 1.0	- 25.7 ± 2.4	2
Sediment										4.9 ±1.2	- 22.2 ± 0.9	
**Ziga**												
*O*. *niloticus*	30	156 ± 28	0.012 ± 0.007	0.27 ± 0.04	83 ± 30	16.7 ± 10.1	6	0.010 ± 0.003	96 ± 4	9.7± 0.6	- 18.4 ± 3.0	1.9 ±0.1
*S*. *membranaceus*	32	228 ± 16	0.164 ± 0.040	0.22 ± 0.06	3 ± 1	1.9 ± 0.4	6	0.142 ± 0.046	81 ± 12	14.4 ± 1.5	- 25.7 ± 0.6	3.3 ± 0.4
*B*. *bajad*	34	293 ± 37	0.101 ± 0.060	0.36 ± 0.06	10 ± 5	5.0 ± 2.0	6	0.094 ± 0.050	83± 17	14.3 ± 0.8	- 20.0 ± 0.7	3.3 ±0.2
*C*. *anguillaris*	31	302 ± 50	0.117 ± 0.090	0.24 ± 0.08	4 ± 3	2.0 ± 1.6	5	0.200 ± 0.060	78 ± 4	12.4 ± 0.7	- 21.7 ± 1.5	2.7 ± 0.2
*S*. *intermedius*	17	146 ± 13	0.102 ± 0.060	0.20 ± 0.05	2 ± 0	1.5 ± 0.2	2	0.082 ± 0.070	86± 12	12.2 ± 0.2	- 21.6 ± 0.8	2.7 ± 0.1
Zooplankton	bulk		0.024		not analysed	not analysed			not analysed	11.0	- 25.3	2
Iridinidae	2		0.040 ± 0.030	0.04 ± 0.06	not analysed	not analysed	2	0.006 ± 0.000	not analysed	11.9 ± 0.5	- 29.5 ± 0.8	2
Gastropoda	3		0.100 ± 0.072	0.30 ± 0.08	not analysed	not analysed	3	0.032 ± 0.006	not analysed	7.3 ± 0.1	- 24.9 ± 0.1	2
Sediment										5.7 ± 1.0	- 19.4 ± 0.9	-
**Kompienga**												
*O*. *niloticus*	20	226 ± 57	0.011 ± 0.010	0.10 ± 0.02	55 ± 22	19 ± 26	6	0.004 ± 0.002	84 ± 27	9.2 ± 0.4	- 18.2 ± 1.0	2.4 ± 0.1
*A*. *occidentalis*	13	298 ± 67	0.074 ± 0.050	0.23 ± 0.43	9 ± 9	4 ± 4.8	8	0.060± 0.032	83 ± 14	10.8 ± 0.9	- 23.8 ± 3.4	2.9 ± 0.3
*S*. *membranaceus*	3	295 ± 85	0.051 ± 0.007	0.19 ± 0.02	8 ± 2	2.9 ± 0.3	3	0.044 ± 0.003	99 ± 7	10.5 ±0.5	- 24.8 ± 1.1	2.8 ± 0.1
*B*. *bajad*	10	438 ± 45	0.213 ± 0.060	0.18 ± 0.03	5 ± 5	2.2 ± 1.7	10	0.176 ± 0.050	100 ± 8	13.2 ± 0.8	- 21.0 ± 1.2	3.6 ± 0.2
*C*. *anguillaris*	13	439 ± 12	0.197 ± 0.080	0.20 ± 0.06	3 ± 2	2.5 ± 1.3	10	0.111 ± 0.070	69 ± 40	11.6 ± 0.7	- 19.9 ± 1.2	3.1 ± 0.2
*L*. *niloticus*	5	364 ± 60	0.190 ± 0.080	0.20 ± 0.02	3 ± 1	1.7 ± 1.2	5	0.150 ± 0.060	77 ± 13	13.7 ± 0.7	- 20.1 ± 0.7	3.7 ± 0.2
*S*. *intermedius*	17	115 ± 5	0.111 ± 0.070	0.15 ± 0.05	2 ± 1	1.4 ± 1.0	7	0.146 ± 0.043	80 ± 25	11.8 ± 1.3	- 21.5 ± 1.5	3.1 ± 0.4
Zooplankton	bulk		0.0078		not analysed	not analysed		0.006	not analysed	10.8	- 25.6	2
Gastropoda	3		0.1 ± 0.072	0.30 ± 0.08	not analysed	not analysed	3	0.032 ± 0.006	not analysed	6.3 ± 0.2	- 21.2 ± 0.3	2
Sediment										5.4 ± 0.2	- 21.0 ± 2.3	-

Abbreviations: n1 is the sample size for THg, TSe analyses, n2 is the selected sample for MeHg analysis. TL refers to total length of fish.

Within each reservoir, THg concentrations varied by two orders of magnitude from an average 6 ng/g w.w. in detritus and invertebrate feeding fish to 230 ng/g w.w. in predatory fish at the top of food web. MeHg concentrations ranged from 6 ng/g w.w. to 185 ng/g w.w. between detritus and invertebrate feeding fish and predatory fish at the top of the food web. Overall, the TSe concentrations did not show significant variation between invertebrates and higher trophic level fish within each reservoir. TSe concentrations in muscle tissues of fish averaged 147 ± 100 ng/g w.w., 264 ± 82 ng/g w.w. and 167 ± 168 ng/g w.w. in Loumbila, Ziga and Kompienga, respectively, which were lower than TSe threshold of 3,000 ng/g w.w.

A stepwise multiple regression analysis using trophic position (δ^15^N), carbon source, and fish size, revealed that δ^15^N was the most significant descriptor explaining THg concentration in fish (Table 3). Fish δ^15^N alone explained 30 and 33% of the variability of THg concentration for Loumbila’s reservoir (F_1,20_ = 8.47, R^2^ = 0.30, p < 0.05) and for Kompienga (F_1,47_ = 23.45, R^2^ = 0.33, p < 0.001). For Ziga reservoir, 45% of the variability of THg concentration in fish was explained by δ^13^C (p < 0.001, F = 10.75, R^2^ = 0.32) followed by total length (Tl) (p < 0.05, F = 7.68, R^2^ = 0.18) (Table 3).

**Table 3:** Relationships between muscleHg and TSe concentrations and fish body size, fish trophic position (δ ^15^N), and carbon source (δ ^13^C) within fish species in the three study reservoirs.

**Metal/Metalloid**	**site**	**n**	**Regression model**	**R** ^2^_adj_	**p-value**
THg	Loumbila	22	log_10_[THg] = 0.30*δ^15^N -7.03	0.30	<0.05
	Ziga	25	[THg] = -0.02*δ^13^C + 0.006*Tl -0.38	0.45	< 0.001
	Kompienga	49	[THg] = 0.04*δ^15^N -0.03	0.33	< 0.001
MeHg	Loumbila	22	log_10_[MeHg] = 0.33* δ^15^N -7.32	0.38	< 0.05
	Ziga	25	[MeHg] = -0.014* δ^13^C + 0.004 *Tl -0.30	0.50	< 0.001
	Kompienga	49	log_10_ [MeHg] = 0.025*δ^15^N -0.001*Tl -0.24	0.48	< 0.001
TSe	Loumbila	22	log_10_[TSe] = a (Tl) + b (δ^15^N) +c (d13C)	0.17	>0.05
	Ziga	25	[TSe] = a (Tl) + b (δ^15^N) +c (d13C)	0.29	>0.05
	Kompienga	49	log_10_ [TSe] = a (Tl) + b (δ^15^N) +c (d13C)	0.05	>0.05

Abbreviations: n is the sample number, Tl refers to total length.

For MeHg concentration in fish, the regression model identified δ^15^N as the main explanatory variable. δ^15^N alone explained 38% of MeHg variation in fish from Loumbila’s reservoir (p < 0.05) whereas, in Kompienga reservoir, δ^15^N followed by fish Tl explained 48% of MeHg concentration in fish. Carbon source was a key variable explaining MeHg concentration in fish from Ziga (p < 0.05, F_1,20_ = 8.87, R^2^ = 0.28) followed by total length (Tl) (p < 0.05, F_1,20_ = 8.48, R^2^ = 0.18) (Table 3).

No significant relationships were reported with TSe concentration and the three regression variables (δ^15^N, δ^13^C and Tl) for the study sites (p > 0.05).

Within fish species from each reservoir, stepwise regression analysis failed to select a variable or a combination of variables among δ^15^N_adj_, δ^13^C values, TSe or THg which would explain mercury (THg and MeHg) or TSe bioaccumulation in fish (S5 Table).

### 3.3 Food chain biomagnification of Hg and Se

Simple linear regressions revealed significant positive relationships between log_10_-Hg (THg and MeHg) concentrations (on a wet weight basis) and δ^15^N values of fish from each reservoir (Table 4; p < 0.05). Based on the fish food web, THg and MeHg biomagnified in the three reservoirs with TMFs ranging from 2.9 (Loumbila) to 6.5 (Kompienga) for THg and from 2.9 (Ziga) to 6.6 (Kompienga) for MeHg (Table 4). THg biomagnification was 2 times more efficient in Kompienga compared to the Ziga and Loumbila reservoirs. In contrast, the relationship of TSe versus δ^15^N was not statistically significant (p > 0.05). The TMF of selenium did not differ from 1 across the study sites (Table 4) indicating constant TSe concentration from the fish at the base of food web to the top fish in each food web. TSe was not biomagnified in these food webs.

**Table 4:** Relationships betweenlog_10-_Metal(loid) concentrationand δ^15^N for THg, MeHg and TSe of fish from three freshwater (Burkina Faso) in rainy season of 2010 and their corresponding trophic magnification factors (TMF).

**Reservoir**	**Regression Equation**	**(Slope ± SD) = TMS**	**R** ^2^	**p- value**	**TMF**
Loumbila (n = 22)	log_10_THg vs δ^15^N	0.13 ± 0.04	0.30	0.008	2.9 ± 1.4
	log_10_ MeHg vs δ^15^N	0.14 ± 0.04	0.38	0.003	3.1 ± 1.4
	log_10_TSe vs δ^15^N	0.02 ± 0.02	0.05	0.290	1
Ziga (n = 25)	log_10_THg vs δ^15^	0.15 ± 0.05	0.30	0.004	3.3 ±1.5
	log_10_ MeHg vs δ^15^N	0.14 ± 0.04	0.28	0.006	2.9 ± 1.4
	log_10_TSe vs δ^15^N	0.01 ± 0.01	0.08	0.168	1
Kompienga (n = 49)	log_10_THg vs δ^15^N	0.23 ± 0.04	0.45	< 0.001	6.5 ±1.3
	log_10_ MeHg vs δ^15^N	0.24 ± 0.03	0.50	< 0.001	6.6 ± 1.3
	log_10_TSe vs δ^15^N	0.04 ± 0.02	0.08	0.046	1.3 ± 1.2

TMF = 10^m^, m = slope × 3.4. Trophic magnification slope (TMS) was given by the slope of the regression betweenlog_10-_Metal(loid) concentrationand δ^15^N, n is the sample number.

**Fig 3:**
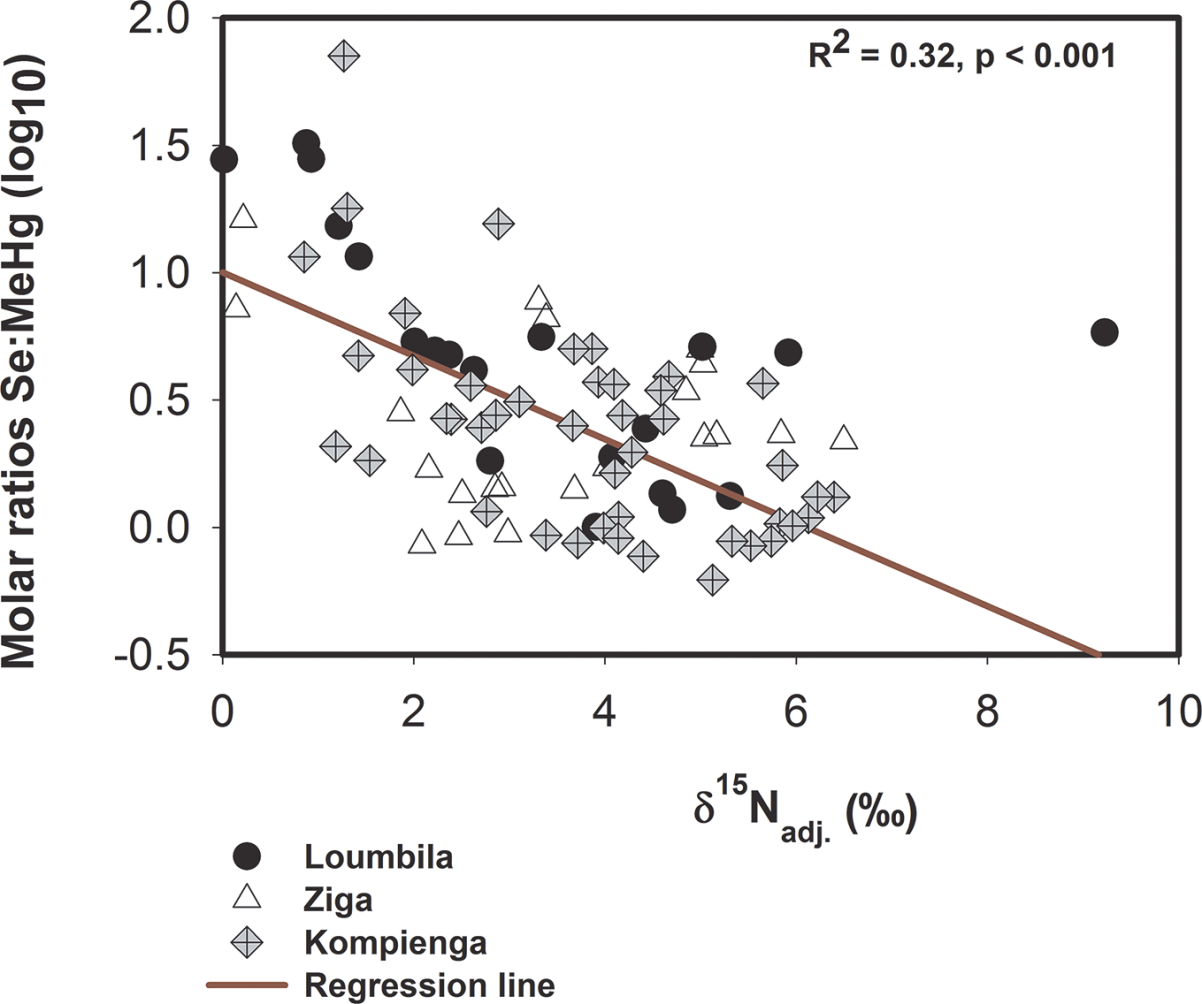
Regression between δ^15^N_adj_ and log-transformed Se:MeHg molar ratios for fish of all reservoirs.

The TSe to THg molar ratios of all fish species collected weregreater than 1 (Table 2) and a significant decrease (p < 0.05) in TSe to MeHg molar ratio with trophic position was observed for all fish species collected in the three reservoirs (Fig 3). This indicated that fish from the reservoirs hadsufficient selenium content to potentially protect them against Hg bioaccumulation and toxicity, although the accumulation of Se compared to MeHg was lower in predatory fish.

### 3.4. Comparison of THg biomagnification and food chain length in fluvial reservoirs with other water bodies in Africa

Most research on Hg biomagnification in African aquatic ecosystems has focused on large (great) lakes (Table 1). The food web TMFs for THg in west African fluvial reservoirs (2.9–6.5) were within the range of values measured in other African systems (2.8–9.0), with an overall TMF average of 4.5 ± 0.5 (1 standard error) (n = 17). Thus, on average, THg biomagnifies by a factor of ~5 between trophic levels in tropical African fresh waters.

**Fig 4:**
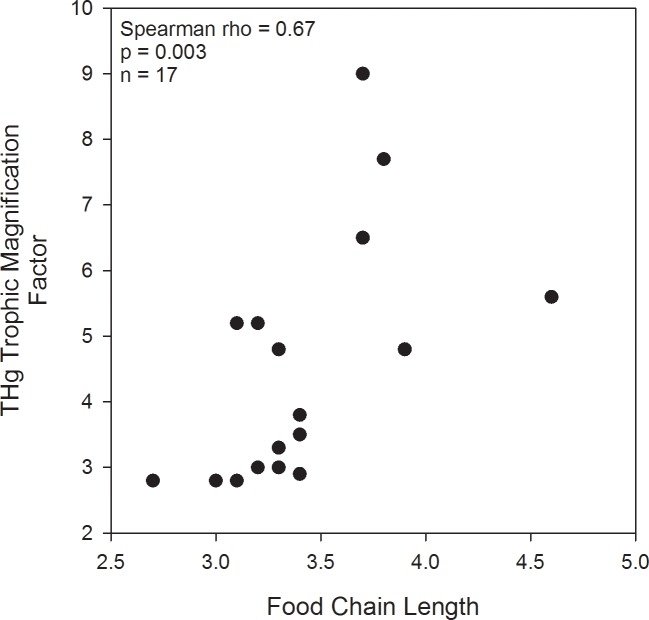
Relationship between the TMF of THg in fish food webs of African water bodies and FCL.

A correlation analysis showed that the variation in TMFs of THg among sites was positively correlated with FCL (Fig 4; Spearman rho = 0.67, p = 0.003, n = 17) but not maximum depth (p = 0.35, n = 16), water body surface area (p = 0.13, n = 16), or the number of fish species included in each study (p = 0.08, n = 17). There was a weak, positive correlation between FCL and the number of fish species sampled (Spearman rho = 0.53, p = 0.027). The two highest FCLs (from Lake Malawi and Lake Tanganyika) were based on single measurements of δ^15^N for the top predator species (S3 Table). When those sites were excluded, the positive correlation between TMF and FCL remained significant (Spearman rho = 0.67, p = 0.005, n = 15). These results showed that THg biomagnified more in lakes with longer food chains, although a positive correlation between the number of fish species sampled and FCL suggests that sampling design may be influencing the among-system differences in the dataset.

**Fig 5:**
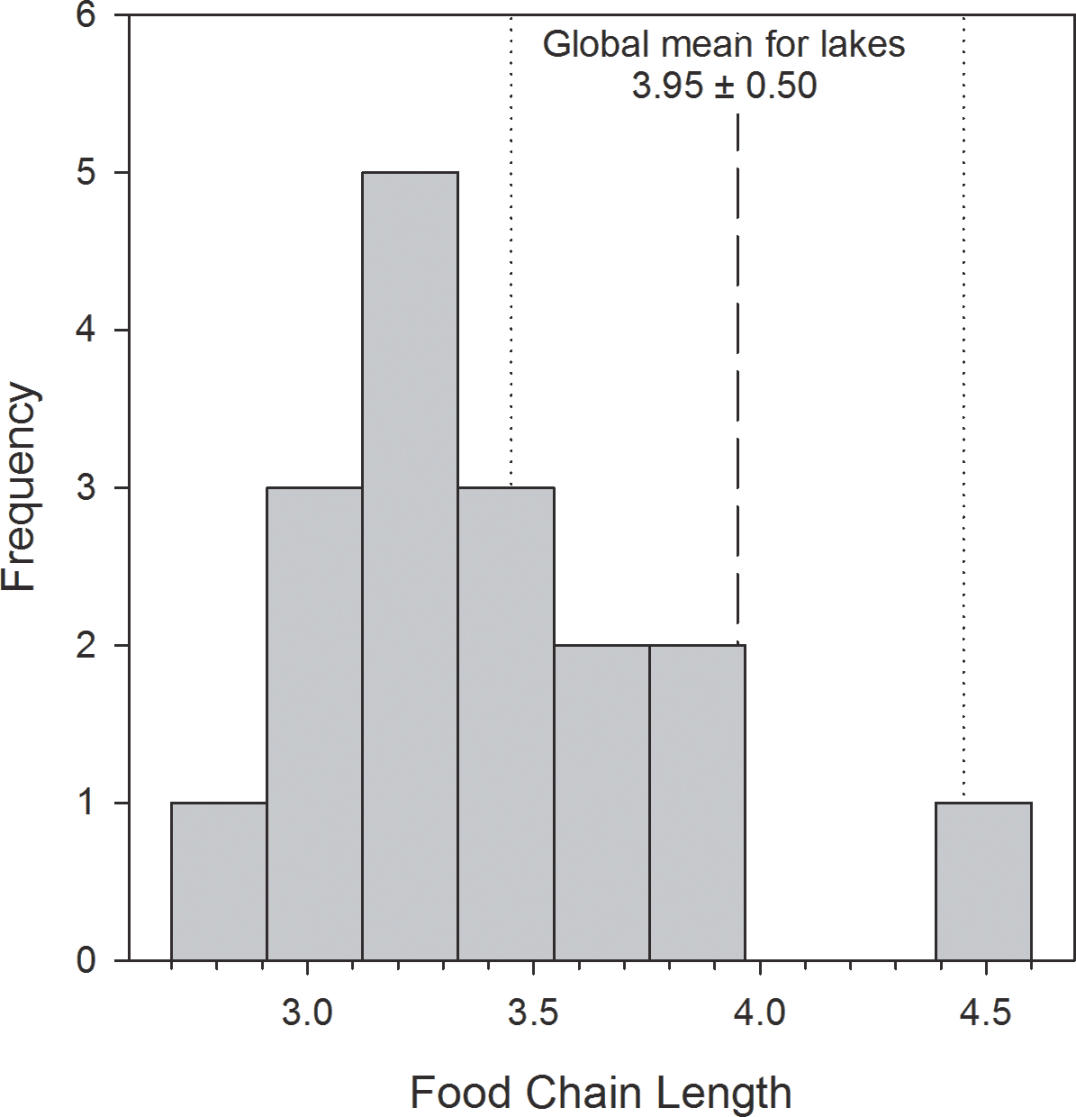
Frequency distribution of FCL measured in THg biomagnification studies for African water bodies. The global mean (± 1 standard deviation) of food chain length in lakes is provided as a reference (Vander Zanden and Fetzer 2007).

The FCLs for the three fluvial reservoirs were relatively low and similar to the average (± 1 standard error) for all sites (3.4 ± 0.1). With the exception of some of the African great lakes, most FCLs in the dataset were shorter than the global mean FCL of 3.95 for lakes (Fig 5; [28]).

The THg concentrations of a commonly sampled fish species (the detrivore *O*. *niloticus*) were similarly low among nine systems (Table 1), ranging from 0.003–0.012 **μ**g/g ww. The average THg concentration of this fish species was not correlated with site TMF (p = 0.80, n = 11). The consistently low THg concentrations in this detrivorous fish suggest similar mercury bioaccumulation occurred lower in the food web (TP = 2.2; this study) of different types of aquatic ecosystems.

## Discussion

### 4.1. Food web structure

The food webs of the three freshwater reservoirs from Burkina Faso had similar structures. Fish were supported by a range of carbon sources and showed large overlap in their δ^15^N values. There was no clear food partitioning among fish from the three reservoirs and this suggests that omnivory and opportunistic feeding habits are common behaviors of fish from these tropical ecosystems, with an estimated 25% of species being omnivores based on their range of δ^15^N values. This was supported by stomach content analysis [32] and by previous studies based on diet analysis of fish from tropical Africa [50,51,52]. A broader spectrum of resources used by fish from these ecosystems justify the approach using mean δ^15^N values of all primary consumers collected, rather than a δ^15^N value of single primary consumer, as a baseline signature to describe and compare food webs structure across reservoirs. It is possible indiscriminate average δ^15^N values ofvarious primary consumers as baseline potentially could lead to erroneous conclusion in trophic position due to variation of invertebrates composition across sites [53]. Likely, baseline variation was not a source of major error in FCL estimates [28]. For δ^13^C, evidence of primary producer δ^13^C fluctuations within and across systems has been reported [35]. Differences in isotopic fractionation during photosynthesis (due to differences in growth rates, CO_2_ availability, biomass) could lead to divergent δ^13^C values for the same carbon source in different reservoirs (or even in the same reservoir at different times or locations). As a result, the significant variation of δ^13^C values of *O*. *niloticus* and *C*. *anguillaris* across the study reservoirs may be due to baseline fluctuation rather than changes in habitat use. The most depleted ^13^C value of *O*. *Niloticus* from Loumbila reservoir could be due to inputs of organic matter from agriculture practice in wetlands surrounding the Loumbila dam.

The FCLs from aquatic systems reported in this study and other water bodies from Africa are lower than the global mean FCL of 3.95 [54,55]. Omnivorous diets of fish in tropical water systems could lead to low FCLs. Alternatively, high levels of omnivory and flexibility in feeding habits are factors that could affect δ^15^N enrichment over time [56]. Uncertainty of the trophic enrichment factor of ^15^N in tropical systems may contribute to the observed pattern in FCL. Because of unavailability of experimentally derived trophic enrichment factors, FCLs have been calculated based on an assumed enrichment of 3.4 ‰ ^15^N per trophic level. If the trophic enrichment of ^15^N was lower than the assumed value of 3.4‰ per trophic level (e.g. due to growth dilution in productive systems), FCLs calculated based on value of 3.4 ‰ would underestimate the real FCLs.

The positive correlation between FCL and TMF (Hg) across African aquatic ecosystems indicates that predatory fish are more contaminated with Hg if they are part of a longer food chain. Therefore, short FCLs could be one possible factor explaining the “lower than expected Hg concentration in fish” generally reported from Africa [17,57,58,59,60].

### 4.2. Factors influencing bioaccumulation and biomagnification of Hg and Se in reservoir systems

Our results showed that trophic position (δ^15^N) and habitat use (carbon signature, δ^13^C) determine Hg concentration in fish tissues as revealed by stepwise multiple regression analysis. A positive and significant relationship between Hg (THg and MeHg) concentration and fish size (Tl) was reported in fish from Ziga’s and Kompienga’s reservoirs, suggesting that Hg bioaccumulation efficiency of these fish were also related to their size. Several studies from tropical localities have shown that Hg concentration in fish generally increases with both size [3,54,61] and trophic position as the result of contaminant accumulation with the exposure time and biomagnification [24,62]. A study carried out in Lake Awassa (Ethiopia) reported the influence of habitat use in the variation of Hg concentration in the piscivorous species *Barbus paludinosus* [63] with fish inhabiting the pelagic zone having higher Hg concentration than those preying on benthic organisms. Habitat use is well known in temperate studies to reflect Hg concentration in organisms in lakes with pelagic dwellers having higher concentrations than littoral organisms due to habitat-specific bioaccumulation of MeHg in prey [38,64,65].

In addition to the influence of trophic position, carbon source and fish total length, we measured very low levels of MeHg in water and of THg in sediments (S1 Table), similar to levels that can be encountered in remote regions with low rates of atmospheric Hg deposition, such as polar lakes [66]. Together, these results suggest a low rate of net MeHg production that may explain low Hg levels measured in animals at the base of the food web. In contrast, TSe concentration in fish did not show any relationship with these three explanatory variables suggesting that others factors may be involved in TSe uptake and bioaccumulation.

The TMF values reported in this study indicated that biomagnification of Hg occurs in aquatic systems of Burkina Faso. The TMF values were consistent with other observations reported from tropical lakes and rivers [3,67,68]. The TMSs of 0.13–0.23 for THg found in Loumbila, Ziga and Kompienga reservoirs were in the range of those reported for other aquatic systems from Africa (0.13–0.28; Table 1) and fish-only food webs globally (0.16 ± 0.13) [69].The processes leading to among site differences in Hg biomagnification rates are not yet well understood [42]. More efficient transfer of THg between trophic levels in lake food webs (measured by TMS values) has been linked with physical and chemical characteristics, particularly nutrient concentrations (or lake trophic status), dissolved organic carbon, and lake size [4,66,69,70,71]. For example, Poste et al. [4], in a recent paper, found a strong negative relationship between THg TMF and trophic status of lakes from Africa (measured as chlorophyl a) providing evidence for the moderating role of eutrophication on Hg biomagnification. Those authors found THg biomagnification occurs at a lower rate in lakes with higher phytoplankton biomass. We found in this study a 2 fold higher biomagnification rate in the Kompienga reservoir compared to the two others reservoirs. Trophic status of these reservoirs was not measured but the smaller volume of Loumbila and Ziga and the widespread practice of agriculture surrounding these dams compared to the large hydroelectric reservoir of Kompienga are consistent with a trophic status explanation. Kompienga reservoir also had a longer FCL consistent with the observation that longer food chains have higher Hg biomagnification. On a global scale, rates of THg biomagnification tend to be greater in fresh waters in polar regions [69].

To our knowledge, little information exists on the influence of FCL on THg biomagnification rate, although Verburg et al. [72] concluded that FCL did not affect the TMS values in three New Zealand lakes. For the present study on African systems, it is unclear what process could lead to greater THg biomagnification in longer food chains. FCL could have a positive influence on THg biomagnification rate if longer food chains support top predator fish that grow to a larger size or older age. Fish length and age are important determinants of Hg bioaccumulation, and larger or older fish for a given trophic level would lead to more efficient trophic transfer of Hg and a higher TMF. Swanson and Kidd [73] found that the TMS was 30% lower in two lakes after the influence of size and age on fish Hg concentration was removed. It is also possible that other variables such as trophic status or lake size may be influencing the trend [70], although we did not find a positive correlation between THg biomagnification rate and lake size in our dataset. Ecosystem size is a key determinant of FCL, and larger lakes have longer food chains [27]. Perhaps the few lakes with long FCL studied in Africa up to now have similar attributes related to aquatic productivity, nutrient inputs or oxygen stratification that may promote higher TMS. Further research is warranted to determine the role of FCL in THg biomagnification.

The results of the linear regression between log_10_TSe and δ^15^N_adj_ show that there is no evidence of TSe biomagnification in the food webs (TMF ~ 1). Biomagnification of Se in food web is still a controversial issue, with some studies reporting an increase with trophic level [5,6,8,74] and others showing the opposite or no clear trends [45,75]. For instance, some studies reported the ability of organic Se concentration of 0.1 **μ**g/L in surface water to biomagnify through food web, reaching higher concentrations (5–15 **μ**g/g) in top predator [74,76]. The chemical forms of Se influence its uptake by algae and microorganisms and its subsequent transfer to upper biota [5,6]. Selenite was identified as the more bioaccumulative form of Se [5].

The higher concentrations of TSe in fish compared to THg reported in this study suggest that these freshwater fish may potentially be protected against Hg bioaccumulation and toxicity. Due to lack of TSe biomagnification and the increase of THg with trophic level, a significant decrease of TSe to MeHg molar ratios with trophic level was observed with all fish collected. This suggests that the protective effect of Se is lesser in top predators, which are also those more contaminated with MeHg.

To our knowledge, this study was the first to examine Se bioaccumulation and biomagnification in freshwater food webs from Africa. This study did not differentiate the forms of selenium in water, but it is known that selenium in the oxidized state (such as those of present study, S1 Fig) is mainly in the form of selenate [5]. Therefore, possible low availability of selenite in our study sites could explain the observed low level of TSe in fish due to dominance of the less bioaccumulative forms of Se (e.g. selenate) in the food chain. We reported a Se to Hg molar ratio greater than 1 in all fish, suggesting fish should be safe for consumption [9,11]. Further, we found an inverse relationship between trophic level and Se:MeHg ratios, indicating that Se has a lesser protective effect in top predators, which are also the most contaminated animals with respect to MeHg. Few studies have examined food web dynamics of Se:MeHg ratios [77], and further studies should target the trophic transfer of Se/Hg complexes, since such complexes are known to detoxify Hg in mammals and fish [8,11,78].

## Conclusion

This study was the first to describe food web structure, the co-occurrence of mercury and selenium bioaccumulation and trophic transfer to fish in West African freshwater reservoirs using stable isotopes analyses. We found relatively low concentrations of Hg and Se in fish. Fish relied on a mixture of pelagic and littoral sources for their diet, with carbon sources influencing their Hg concentration. We also found short FCLs in the reservoirs compared to some African great lakes and to the global average for lakes. Fish Hg concentrations were relatively low in the three reservoirs, which likely reflects a combination of factors: 1) low levels of MeHg in water and of THg in sediments leading to low MeHg uptake at the base of the food webs (as indicated by the low THg concentrations in detritivorous fish); 2) the low trophic position of many fish species in these systems; and 3) less efficient biomagnification of Hg in systems with shorter food chains. Selenium did not biomagnify in the reservoirs, in contrast with Hg, and as a result, Hg:Se ratios declined with increasing trophic position of fish, although always was >1, The mechanisms controlling biomagnification rates in African foodwebs remains unclear. Further research is needed to improve our understanding on Hg and Se biogeochemical cycles and the processes leading to lower bioaccumulation in African freshwater ecosystems.

## Supporting Information

S1 Fig Water column physicochemical profiles of the study sites. Water temperature (T) and Dissolved oxygen (DO). Bottom waters were well oxygenated (range 20–100%). None of the sites were stratified.
(DOCX)

S1 Protocol Details on protocols for mercury and selenium analyses and references for supplementary information section. (DOCX)

S1 Table Environmental characteristics of the study sites during the 2010 rainy season. (DOCX)

S2 Table Quality of analytical results of metal(loïd) in water and fish tissues. DORM-2, DORM-3, TORT-2 are certified reference materials (CRM) from the National Research Council of Canada. (DOCX)

S3 Table Description of the biota used to estimate FCLs in African water bodies. In most cases, the site-specific baseline δ^15^N value was estimated using the mean of all data available for primary consumer invertebrates from the study although for two sites, the mean of data for primary producers (benthic algae and phytoplankton) was used. The fish species with the highest mean δ^15^N in each study was used for the top predator.
(DOCX)

S4 Table Isotopes ratios of δ13C and δ15N and TP of biota collected in freshwater from Burkina Faso during the rainy season of 2010. (DOCX)

S5 Table Relationships between Hg, TSe and fish trophic position, carbon source and co-occuring metal(loïd) concentration. (DOCX)
