# Supplementary material for: Bioaccumulation and Trophic Transfer of Mercury and Selenium in African Sub-Tropical Fluvial Reservoirs Food Webs (Burkina Faso)
Source: PLoS One. 2015 Apr 13;10(4):e0123048. doi: 10.1371/journal.pone.0123048 (PMC4395242; doi:10.1371/journal.pone.0123048)
Supplement: S1 Fig — Water temperature (T) and Dissolved oxygen (DO). Bottom waters were well oxygenated (range 20–100%). None of the sites were stratified. (DOCX) [file pone.0123048.s001.docx]

**
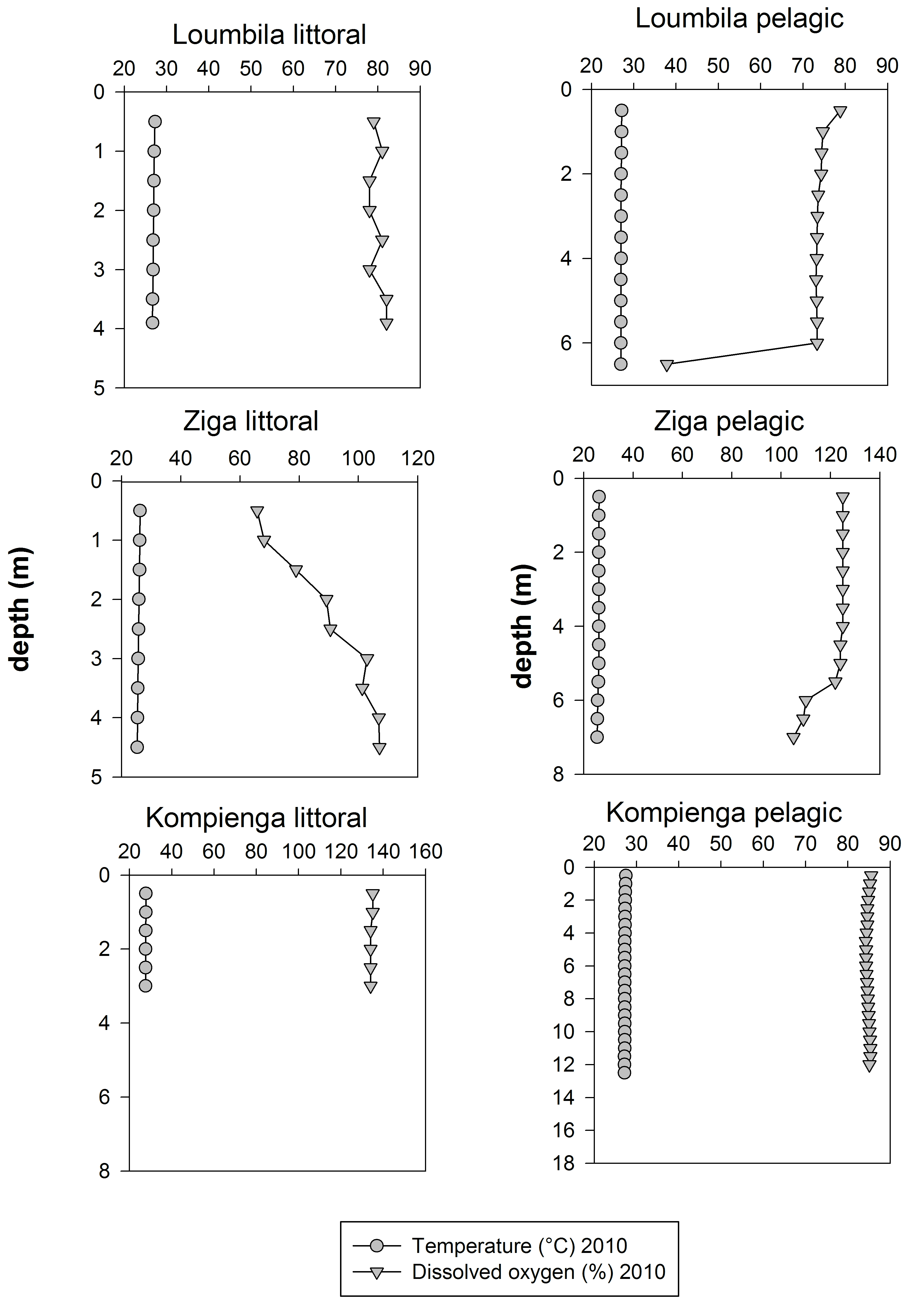
**

**S1 Figure. Physicochemical profiles of the study sites**. Water temperature (T), Dissolved oxygen (DO). Bottom waters were well oxygenated (range 20 -100%). None of the sites were stratified.
