# Supplementary material for: Bioaccumulation and Trophic Transfer of Mercury and Selenium in African Sub-Tropical Fluvial Reservoirs Food Webs (Burkina Faso)
Source: PLoS One. 2015 Apr 13;10(4):e0123048. doi: 10.1371/journal.pone.0123048 (PMC4395242; doi:10.1371/journal.pone.0123048)
Supplement: S1 Protocol — (DOCX) [file pone.0123048.s002.docx]

**S1** **Protocol.** **Details on protocol for mercury and selenium analyses and references for supplementary information section.**

1. **Water sample collection**

Ultra clean protocols for trace metals [1] were employed to collect water. In each reservoir, water was collected on one occasion at the near shore station (littoral zone) and open water station (pelagic zone) at 0.5 m from the sediment surface where THg and MeHg concentrations were potentially higher [2]. Water was collected with a peristaltic pump and acid-washed Teflon tubing. Triplicates of filtered and unfiltered water samples for THg and MeHg were collected and stored in 125 mL amber glass bottles that had been pre-washed with acid and thoroughly rinsed with ultrapure water (Milli-Q: > 18 Mohm cm^-1^) and placed in double ziplock bags for transport to the field. Filtration was done onboard using a Whatman syringe filter of 0.45 µm pore size. All bottles were rinsed three times with dam water prior to water collection. All aqueous mercury samples were preserved at pH 2 with ultra high purity hydrochloric acid (VWR) (0.5%, v/v) and kept in a field cooler and refrigerated (+ 4 °C) upon return to laboratory until analysis. Samples for ancillary chemical analyses were also collected at the same depth. Filtered water was collected in two separate 30 mL Nalgene HDPE bottles. One was acidified using hydrochloric acid (0.5%, v/v) for major cations [potassium (K^+^), calcium (Ca^2+^), sodium (Na^+^) and magnesium (Mg^2+^)], the second was left unacidified for analysis of anions [chloride (Cl^-^), nitrite-nitrate (NO_3_^-^), sulphate (SO_4_^2-^)]. For dissolved organic carbon (DOC) analyses, water samples were filtered on 0.45 µm Whatman filters and collected in glass bottles (pre-heated at 550 °C during 1h). Filtered water samples for selenium analysis were collected in 30 mL Nalgene HDPE bottle samples and preserved with 1% v/v EDTA [3].

1. **Water analyses**
   1. **THg analysis**

THg analysis in water samples (filtered and unfiltered) was performed by cold vapor atomic fluorescence spectrometer (CVAFS, Tekran 2600, Tekran Instruments Corporation, Knoxville, TN, USA) following U.S. Environmental Protection Agency (U.S. EPA) method 1631. Briefly, 50 mL of sample was digested with 200 µL of BrCl, and excess of BrCl was neutralized with 50 *μ*L of hydroxylamine. Samples were then reduced with stannous chloride (SnCl_2,_3% w/v) prior to analysis. The detection limit for this analysis was 0.13 ng THg/L and the mean relative recovery was 104 ± 5 % (n=5). The coefficient of variation (standard deviation/mean) for field triplicate determinations was 2%.

- 1. **Methylmercury analysis.**

Water samples (50 mL) for MeHg were acid-distilled to remove matrix interferences, then derivatized by aqueous-phase ethylation with NaB(C_2_H_5_)_4_, purged on Tenax (Tenax Corporation, Baltimore, MD, USA), separated by gas chromatography and quantified with a Tekran 2500 CVAFS (Tekran Instruments Corporation) based on the method of Bloom [4]. Field and procedural blanks contained less than 1 ± 1 pg MeHg and revealed no contamination during sampling, filtration, distillation, and analysis. Analytical accuracy was checked by analysis of TORT-2 (S2 Table)

- 1. **Selenium analysis**

Selenium determination protocol is the same as in Ouédraogo and Amyot [5]. Prior to total selenium analysis in water samples, 4 mL of water samples were digested in acid mixture of HCl (4 mL) and HNO_3_ (0.48 mL) to allow the reduction of Se (VI) to Se (IV). NaBH_4_ (1.1 % m/v in 0.1M NaOH v/v) was added to the digested samples to produce hydrides and the levels of TSe were determined by Hydride Generation Atomic Fluorescence Spectrometry (HG-AFS, PSA 10.055, Millenium Excalibur; PS Analytical, Orpington, Kent, UK). For TSe determination in solid samples (fish, zooplankton, gastropods and bivalves), 20 to 50 mg of solid tissues were submitted to microwave digestion with a mixture of HNO_3_ and H_2_O_2_ based on a method developed by Corns et al. [6] in order to extract elements from solid matrix. An aliquot was then taken and underwent the same steps as for aqueous samples.

The analytical quality was controlled by using certified reference materials DORM-3 and TORT-2 from the National Research Council of Canada (S2 Table). Efficacy of Se (VI) conversion to Se (IV) was checked by using a solution of Se (VI) which was analyzed together with the samples. Procedural blanks were 21 ± 8 ng.L^-1^ (n=8). The method detection limit (MDL) was 22 ng L^-1^(aqueous Se) and 0.022 µg/g dry weight (d.w.) for solid samples. Se (VI) (200 ng.L^-1^) conversion to Se (IV) averaged 109 % ± 9.

- 1. **Other physico-chemical analyses**

Anions (Cl^-^, SO_4_^2-^, NO_3_^-^) were analysed by ion chromatography using a DIONEX-DX500 (MDLs: 1 µmol L^-1^ for the three ions). Cations (Ca^2+^, Mg^2+^, K^+^ and Na+) were analysed by atomic absorption spectrometry with MDLs of 0.5, 0.1, 0.5 and 0.5 µmol L^-1^, respectively.

1. U.S.E.P.A. Method 1669: Sampling Ambient Water for Trace Metals at EPA Water Quality Criteria Levels. Washington, D.C. 1996; 20460.

2. Morel FMM, Kraepiel AML, Amyot M. The chemical cycle and bioaccumulation of mercury. Annu Rev Ecol Syst. 1998;29: 543-566.

3. Bednar AJ, Garbarino JR, Ranville JF, Wildeman TR. Preserving the distribution of inorganic arsenic species in groundwater and acid mine drainage samples. Environ Sci Technol. 2002;36: 2213-2218.

4. Bloom NS. Determination of picogram levels of methylmercury by aqueous phase ethylation, followed by cryogenic gas chromatography with cold vapour atomic fluorescence detection. Can J Fish Aquat Sci. 1989;46: 1131-1140.

5. Ouédraogo O, Amyot M. Mercury, arsenic and selenium concentrations in water and fish from sub-Saharan semi-arid freshwater reservoirs (Burkina Faso). Sci Total Environ. 2013;444: 243-254.

6. Corns WT, Stockwell PB, Ebdon L, Hill SJ. Development of an Atomic Fluorescence Spectrometer for the Hydride forming Elements. J Anal Atom Spectrom. 1993;8: 71-77.

7. Campbell LM, Hecky RE, Nyaundi J, Muggide R, Dixon DG. Distribution and food-web transfer of mercury in Napoleon and Winam Gulfs, Lake Victoria, East Africa. J Great Lakes Res. 2003;29: 267-282.

8. Poste AE, Muir DCG, Mbabazi D, Hecky RE. Food web structure and mercury trophodynamics in two contrasting embayments in northern Lake Victoria J Great Lakes Res. 2012;38: 699-707.

9. Campbell LM, Balirwa JS, Dixon DG, Hecky RE. Biomagnification of mercury in fish from Thruston Bay, Napoleon Gulf, Lake Victoria (East Africa). Africa J Aqua Sci. 2004;29: 91-96.

10. Campbell LM, Wandera SB, Thacker RJ, Dixon DG, Hecky RE. Trophic niche segregation in the Nilotic ichthyofauna of Lake Albert (Uganda, Africa) Environ. Biol Fish. 2005;74: 247-260.

11. Kidd KA, Bootsma HA, Hesslein RH, Lockhart WL, Hecky RE. Mercury concentrations in the food web of Lake Malawi, East Africa. J Great Lakes Res. 2003;29: 258-266.

12. Campbell L, Verburg P, Dixon DG, Hecky RE. Mercury biomagnification in the food web of Lake Tanganyika (Tanzania, East Africa). Sci Total Environ. 2008;402: 184-191.

13. Campbell L, Hecky RE, Dixon DG, Chapman LJ. Food web structure and mercury transfer in two contrasting Ugandan highland crater lakes (East Africa). Africa J Ecol. 2006;44: 337-346.

14. Kidd KA, Stern G, Lemoalle J. Mercury and other contaminants in fish from Lake Chad, Africa. B Environ Contam Tox. 2004;73: 249-256.

15. Tadiso TM, Borgstrøm R, Rosseland BO. Mercury concentrations are low in commercial fish species of Lake Ziway, Ethiopia, but stable isotope data indicated biomagnification. Ecotox Environ Safe 2011;74: 953-959.

16. Poste AE, Hecky RE, Muir D. Biomagnification of mercury in a West African crater lake (Lake Bosomtwe, Ghana). International Association of Theoretical and Applied Limnology, Vol 30, Pt 4, Proceedings 2008;30: 647-650.
