# Supplementary material for: Bioaccumulation and Trophic Transfer of Mercury and Selenium in African Sub-Tropical Fluvial Reservoirs Food Webs (Burkina Faso)
Source: PLoS One. 2015 Apr 13;10(4):e0123048. doi: 10.1371/journal.pone.0123048 (PMC4395242; doi:10.1371/journal.pone.0123048)
Supplement: S1 Table — (DOCX) [file pone.0123048.s003.docx]

**S1 Table**. **Some environmental characteristics in littoral and pelagic areas of the three study sites (Loumbila, Ziga and Kompienga) during 2010 rainy season**.

| **Site** |  |  | **Loumbila** | |  | **Ziga** | |  | **Kompienga** | |
| --- | --- | --- | --- | --- | --- | --- | --- | --- | --- | --- |
| **Compartiment** | variable |  | Littoral | Pelagic |  | Littoral | Pelagic |  | Littoral | Pelagic |
| **water** |  |  |  |  |  |  |  |  |  |  |
|  | Depth (m) |  | 3.5 | 6 |  | 4 | 6.5 |  | 2.5 | 12 |
|  | NO_3_^-^ (mg/L) |  | 1.036 | 1.057 |  | 0.508 | 0.338 |  | 0.447 | 0.341 |
|  | SO_4_^2-^(mg/L) |  | 0.593 | 0.558 |  | 0.773 | 0.736 |  | 0.725 | 0.7 |
|  | DOC (mg/L) |  | 2.53 | 1.59 |  | 2.39 | 1.49 |  | 2.08 | 1.62 |
|  | Conductivity (µS/cm) |  | 82 | 81 |  | 103 | 109 |  | 134 | 134 |
|  | T (ᵒ C) |  | 26.7 | 26.93 |  | 25.36 | 25.57 |  | 27.6 | 27.17 |
|  | DO (%) |  | 73 | 73.3 |  | 106.9 | 96 |  | 88.2 | 85.1 |
|  | pH |  | 7.25 | 7.25 |  | 7.58 | 7.79 |  | 7.07 | 7.32 |
|  | THg (ng/L) |  | 3 | 3.04 |  | 4.93 | 5.42 |  | 2.38 | 2.48 |
|  | DHg (ng/L) |  | 2.5 | 2.5 |  | 1.92 | 2.03 |  | 1.52 | 1.57 |
|  | MeHg (ng/L) |  | 0.037 | 0.11 |  | 0.065 | 0.196 |  | 0.039 | 0.042 |
|  | Tse (ng/L) |  | 72.7 | 65.4 |  | _ | 62.5 |  | 57.5 | 55.8 |
| **sediment** |  |  |  |  |  |  |  |  |  |  |
|  | THg (ng/g; d.w) |  | 7.87 | 19.7 |  | 13.74 | 27.24 |  | 19.11 | 27.19 |
|  | Tse (ng/g; d.w) |  | 62 | 255 |  | 178 | 335 |  | 173 | 142 |
