# Supplementary material for: Bioaccumulation and Trophic Transfer of Mercury and Selenium in African Sub-Tropical Fluvial Reservoirs Food Webs (Burkina Faso)
Source: PLoS One. 2015 Apr 13;10(4):e0123048. doi: 10.1371/journal.pone.0123048 (PMC4395242; doi:10.1371/journal.pone.0123048)
Supplement: S2 Table — (DOCX) [file pone.0123048.s004.docx]

**S2 Table. Quality of analytical results of metal(loïd) in water and fish tissues.** DORM-2, DORM-3, TORT-2 are certified reference materials (CRM) from the National Research Council of Canada. Sample size (n)

| **Element** | **TORT-2 (ng.g-1)** | | | | **DORM-2 (Hg), DORM-3 (As,Se) (ng.g-1)** | | | |
| --- | --- | --- | --- | --- | --- | --- | --- | --- |
|  | Certified value | Our results | Sample (n) | Recovery  (%) | Certified value | Our results | Sample (n) | Recovery (%) |
| THg | 300 ± 15 | 297,25 ±10 | 39 | 99 ± 3 | 4640 ± 260 | 3233 ± 526 | 29 | 70 ± 11 |
| MeHg | 152 ± 13 | 160 ± 22 | 9 | 105 ± 14 | _ | _ | _ | _ |
| TSe | 5630 ± 600 | 5225 ± 414 | 17 | 93 ± 7 | 3300 ± | 3370 ± 230 | 18 | 91 ± 7 |
