# Supplementary material for: Bioaccumulation and Trophic Transfer of Mercury and Selenium in African Sub-Tropical Fluvial Reservoirs Food Webs (Burkina Faso)
Source: PLoS One. 2015 Apr 13;10(4):e0123048. doi: 10.1371/journal.pone.0123048 (PMC4395242; doi:10.1371/journal.pone.0123048)
Supplement: S3 Table — In most cases, the site-specific baseline δ15N value was estimated using the mean of all data available for primary consumer invertebrates from the study although for two sites, the mean of data for primary producers (benthic algae and phytoplankton) was used. The fish species with the highest mean δ15N in each study was used for the top predator. (DOCX) [file pone.0123048.s005.docx]

**S3 Table**. **Description of the biota used to estimate food chain length in African water bodies, their δ^15^N values, sample sizes and taxonomic names.**

| **Water body** | **Type** | **Baseline δ^15^N (‰)** | | **Top predator δ^15^N (‰)** | | **Food chain length** | **Reference** |
| --- | --- | --- | --- | --- | --- | --- | --- |
|  |  | **δ^15^N (*n*)** | **Biota** | **δ^15^N (*n*)** | **Biota** |  |  |
| Victoria (Napoleon Bay) | great lake | 3.8 (5) | *Caridina nilotica* | 8.5 (9) | *Lates niloticus* (>20 cm length) | 3.4 | Campbell et al. 2003 |
| Victoria (Napoleon Gulf) | great lake | 4.9 (5) | zooplankton, chironomids | 9.3 (1) | *Bagrus docmac* | 3.3 | Poste et al. 2012 |
| Victoria (Winham Bay) | great lake | 7.3 (6) | bivalve, ephemeroptera | 12.0 (13) | *Lates niloticus* (>20 cm length) | 3.4 | Campbell et al. 2003 |
| Victoria (Murchison Bay) | great lake | 6.6 (5) | zooplankton, snails, mayflies, chironomids | 10.5 (9) | *Protopterus*  *aethiopicus* | 3.1 | Poste et al. 2012 |
| Victoria (Thruston Bay) | great lake | 3.2 (1) | *Caridina nilotica* | 8.9 (9) | *Lates niloticus* | 3.7 | Campbell et al. 2004 |
| Albert | great lake | 5.5 (13) | *Caridina nilotica,* plecoptera, zooplankton | 11.6 (22) | *Hydrocynus forskahlii* | 3.8 | Campbell et al. 2005 |
| Malawi | great lake | 2.7 (15) | gastropod, bivalve, sponge, zooplankton, | 9.1 (1) | *Clarius* sp. | 3.9 | Kidd et al. 2003 |
| Tanganyika | great lake | 0.3 (20) | benthic algae, phytoplankton | 12.6 (1) | *Polypterus congicus* | 4.6 | Campbell et al. 2008 |
| Saka | lake | -1.6 (2) | benthic algae, phytoplankton | 6.4 (9) | *Lates niloticus* | 3.3 | Campbell et al. 2006 |
| Nkuruba | lake | 3.5 (2) | gastropod, chironomid | 7.7 (5) | *Poecilia reticulata* | 3.2 | Campbell et al. 2006 |
| Chad | lake | 6.4 (5) | oyster (*Etheria elliptica*) | 10.2 (4) | *Clarias gariepinus* | 3.1 | Kidd et al. 2004 |
| Ziway | lake | 7.9 (2) | gastropod | 10.2 (24) | *Clarias gariepinus* | 2.7 | Tadiso et al. 2011 |
| Bosomtwe | lake | 12.0 (12) | zooplankton | 15.3 (5) | *Hemichromis fasciatus* | 3.0 | Poste et al. 2008 |
| Abrewe | river estuary | 10.1 (4) | invertebrates including chironomids | 14.2 (3) | *Chromidotilapia güntheri* | 3.2 | Poste et al. 2008 |
| Loumbila | fluvial reservoir | 7.9 (7) | gastropod, zooplankton, bivalve (Iridinidae) | 12.8 (3) | *Schilbe intermedius* | 3.4 | this study |
| Ziga | fluvial reservoir | 9.9 (7) | gastropod, zooplankton, bivalve (Iridinidae) | 14.4 (6) | *Synodontis membranaceus* | 3.3 | this study |
| Kompienga | fluvial reservoir | 7.8 (5) | gastropod, zooplankton | 13.7 (5) | *Lates niloticus* | 3.7 | this study |

In most cases, the site-specific baseline δ^15^N value was estimated using the mean of all data available for primary consumer invertebrates from the study although for two sites, the mean of data for primary producers (benthic algae and phytoplankton) was used. The fish species with the highest mean δ^15^N in each study was used for the top predator.
