# Supplementary material for: Bioaccumulation and Trophic Transfer of Mercury and Selenium in African Sub-Tropical Fluvial Reservoirs Food Webs (Burkina Faso)
Source: PLoS One. 2015 Apr 13;10(4):e0123048. doi: 10.1371/journal.pone.0123048 (PMC4395242; doi:10.1371/journal.pone.0123048)
Supplement: S4 Table — (DOCX) [file pone.0123048.s006.docx]

**S4 Table. Isotope ratios of δ^13^C and δ^15^N** **and TP of biota collected in three freshwater reservoirs (Loumbila, Ziga and Kompienga) from Burkina Faso during the rainy season of 2010.**

| **Reservoir/Organism** | **(n)** | **δ^15^N (‰)** | **δ ^13^C (‰)** | **TP** |
| --- | --- | --- | --- | --- |
| **Loumbila** |  |  |  |  |
| *O. niloticus* | (5) | 8.8 ± 0.5 | - 27.4 ± 0.7 | 2.2 ±0.1 |
| *A. occidentalis* | (5) | 10.9 ± 1.0 | - 25.9 ± 1.4 | 2.9 ± 0.3 |
| *C. anguillaris* | (9) | 12.1 ± 2.2 | - 23.0 ± 2.0 | 3.2 ± 0.6 |
| *S. intermedius* | (3) | 12.8 ± 0.4 | - 24.1 ± 1.0 | 3.4 ± 0.1 |
| Zooplankton | (bulk) (2) | 10.9 ± 0.18 | -25.4 ± 0.2 | 2 |
| Iridinidae | (2) | 10.0 ± 0.2 | - 28.3 ± 0.1 | 2 |
| Gastropod | (3) | 5.5 ± 1.0 | - 25.7 ± 2.4 | 2 |
| Sediment |  | 4.9 ±1.2 | - 22.2 ± 0.9 |  |
| **Ziga** |  |  |  |  |
| *O. niloticus* | (5) | 9.7± 0.6 | - 18.4 ± 3.0 | 1.9 ±0.1 |
| S. membranaceus | (6) | 14.4 ± 1.5 | - 25.7 ± 0.6 | 3.3 ± 0.4 |
| *B. bajad* | (6) | 14.3 ± 0.8 | - 20.0 ± 0.7 | 3.3 ±0.2 |
| *C. anguillaris* | (6) | 12.4 ± 0.7 | - 21.7 ± 1.5 | 2.7 ± 0.2 |
| *S. intermedius* | (2) | 12.2 ± 0.2 | - 21.6 ± 0.8 | 2.7 ± 0.1 |
| Zooplankton | (bulk) | 11.0 | - 25.3 | 2 |
| Iridinidae | (2) | 11.9 ± 0.5 | - 29.5 ± 0.8 | 2 |
| Gastropoda | (3) | 7.3 ± 0.1 | - 24.9 ± 0.1 | 2 |
| Sediment | - | 5.7 ± 1.0 | - 19.4 ± 0.9 | - |
| **Kompienga** |  |  |  |  |
| *O. niloticus* | (6) | 9.2 ± 0.4 | - 18.2 ± 1.0 | 2.4 ± 0.1 |
| *A. occidentalis* | (8) | 10.8 ± 0.9 | - 23.8 ± 3.4 | 2.9 ± 0.3 |
| S. membranaceus | (2) | 10.5 ±0.5 | - 24.8 ± 1.1 | 2.8 ± 0.1 |
| *B. bajad* | (6) | 13.2 ± 0.8 | - 21.0 ± 1.2 | 3.6 ± 0.2 |
| *C. anguillaris* | (9) | 11.6 ± 0.7 | - 19.9 ± 1.2 | 3.1 ± 0.2 |
| *L. niloticus* | (5) | 13.7 ± 0.7 | - 20.1 ± 0.7 | 3.7 ± 0.2 |
| *S. intermedius* | (8) | 11.8 ± 1.3 | - 21.5 ± 1.5 | 3.1 ± 0.4 |
| Zooplankton | (bulk) | 10.8 | - 25.6 | 2 |
| Gastropoda | (3) | 6.3 ± 0.2 | - 21.2 ± 0.3 | 2 |
| Sediment | - | 5.4 ± 0.2 | - 21.0 ± 2.3 | - |

Sample sizes (n) are in parentheses. Abbreviations: C is carbon, N is nitrogen and TP refers to trophic position.
