# Supplementary material for: Bioaccumulation and Trophic Transfer of Mercury and Selenium in African Sub-Tropical Fluvial Reservoirs Food Webs (Burkina Faso)
Source: PLoS One. 2015 Apr 13;10(4):e0123048. doi: 10.1371/journal.pone.0123048 (PMC4395242; doi:10.1371/journal.pone.0123048)
Supplement: S5 Table — (DOCX) [file pone.0123048.s007.docx]

**S5 Table.** **Relationships between Hg, TSe and fish trophic position, carbon source and co-occuring metal(loïd) concentration.**

| Metal/Metalloid | Fish Species | Reservoir | n | Regression model | R^2^ | p-value |
| --- | --- | --- | --- | --- | --- | --- |
| THg | ***O. niloticus*** | Loumbila | 5 | [THg] = aXδ ^15^N_adj + bX_δ ^13^C + cx[Tse] |  | >0.05 |
|  |  | Ziga | 5 | [THg] = aXδ ^15^N_adj + bX_δ ^13^C + cx[Tse] |  | >0.05 |
|  |  | Kompienga | 6 | [THg] = aXδ ^15^N_adj + bX_δ ^13^C + cx[Tse] |  | >0.05 |
|  |  |  |  |  |  |  |
|  | ***C. anguillaris*** | Loumbila | 6 | [THg] = aXδ ^15^N_adj + bX_δ ^13^C + cx[Tse] |  | >0.05 |
|  |  | Ziga | 6 | [THg] = aXδ ^15^N_adj + bX_δ ^13^C + cx[Tse] |  | >0.05 |
|  |  | Kompienga | 9 | [THg] = aXδ ^15^N_adj + bX_δ ^13^C + cx[Tse] |  | >0.05 |
|  |  |  |  |  |  |  |
|  | ***A occidentalis*** | Loumbila | 5 | [THg] = aXδ ^15^N_adj + bX_δ ^13^C + cx[Tse] |  | >0.05 |
|  |  | Kompienga | 9 | [THg] = _-0.008 X_ δ ^13^C -0.11 | 0.74 | =0.002 |
|  |  |  |  |  |  |  |
|  | ***B. bajad*** | Ziga | 6 | [THg] = aXδ ^15^N_adj + bX_δ ^13^C + cx[Tse] |  | >0.05 |
|  |  | Kompienga | 6 | [THg] = aXδ ^15^N_adj + bX_δ ^13^C + cx[Tse] |  | >0.05 |
|  |  |  |  |  |  |  |
| MeHg | ***O. niloticus*** | Loumbila | 5 | [MeHg] = aXδ ^15^N_adj + bX_δ ^13^C + cx[Tse] |  | >0.05 |
|  |  | Ziga | 5 | [MeHg] = aXδ ^15^N_adj + bX_δ ^13^C + cx[Tse] |  | >0.05 |
|  |  | Kompienga | 6 | [MeHg] = aXδ ^15^N_adj + bX_δ ^13^C + cx[Tse] |  | >0.05 |
|  |  |  |  |  |  |  |
|  | ***C. anguillaris*** | Loumbila | 6 | [MeHg] = aXδ ^15^N_adj + bX_δ ^13^C + cx[Tse] |  | >0.05 |
|  |  | Ziga | 6 | [MeHg] = aXδ ^15^N_adj + bX_δ ^13^C + cx[Tse] |  | >0.05 |
|  |  | Kompienga | 9 | [MeHg] = aXδ ^15^N_adj + bX_δ ^13^C + cx[Tse] |  | >0.05 |
|  |  |  |  |  |  |  |
|  | ***A occidentalis*** | Loumbila | 5 | [MeHg] = _0.012 X_ δ ^13^C +0.34 | 0.96 | =0.003 |
|  |  | Kompienga | 9 | [MeHg] = aXδ ^15^N_adj + bX_δ ^13^C + cx[Tse] |  | >0.05 |
|  |  |  |  |  |  |  |
|  | ***B.bajad*** | Ziga | 6 | [MeHg] = aXδ ^15^N_adj + bX_δ ^13^C + cx[Tse] |  | >0.05 |
|  |  | Kompienga | 6 | [MeHg] = aXδ ^15^N_adj + bX_δ ^13^C + cx[Tse] |  | >0.05 |
|  |  |  |  |  |  |  |
| TSe | ***O. niloticus*** | Loumbila | 5 | [TSe] = aXδ ^15^N_adj + bX_δ ^13^C + cx[THg] |  | >0.05 |
|  |  | Ziga | 5 | [TSe] = aXδ ^15^N_adj + bX_δ ^13^C + cx[THg] |  | >0.05 |
|  |  | Kompienga | 6 | [TSe] = aXδ ^15^N_adj + bX_δ ^13^C + cx[THg] |  | >0.05 |
|  |  |  |  |  |  |  |
|  | ***C. anguillaris*** | Loumbila | 6 | [TSe] = aXδ ^15^N_adj + bX_δ ^13^C + cx[THg] |  | >0.05 |
|  |  | Ziga | 6 | [TSe] = aXδ ^15^N_adj + bX_δ ^13^C + cx[THg] |  | >0.05 |
|  |  | Kompienga | 9 | [TSe] = aXδ ^15^N_adj + bX_δ ^13^C + cx[THg] |  | >0.05 |
|  |  |  |  |  |  |  |
|  | **B. bajad** | Ziga | 6 | [TSe] = aXδ ^15^N_adj + bX_δ ^13^C + cx[THg] |  | >0.05 |
|  |  | Kompienga | 6 | [TSe] = aXδ ^15^N_adj + bX_δ ^13^C + cx[THg] |  | >0.05 |
|  |  |  |  |  |  |  |
|  | ***A occidentalis*** | Loumbila |  | [TSe] = aXδ ^15^N_adj + bX_δ ^13^C + cx[THg] |  | >0.05 |
|  |  | Kompienga |  | [TSe] = aXδ ^15^N_adj + bX_δ ^13^C + cx[THg] |  | >0.05 |

n is the number of fish
